# On the ‘Supersolid’ Response of the Second Layer of $^4 \hbox {He}$ on Graphite

**DOI:** 10.1007/s10909-017-1779-x

**Published:** 2017-04-27

**Authors:** J. Nyéki, A. Phillis, B. Cowan, J. Saunders

**Affiliations:** Department of Physics, Royal Holloway University of London, Egham, Surrey, TW20 0EX UK

**Keywords:** Superfluid Helium-4, Film, Supersolid

## Abstract

Recent torsional oscillator measurements on the second layer of $^4 \hbox {He}$ adsorbed on graphite have identified an anomalous superfluid response over a coverage range near third-layer promotion, with four distinct coverage regimes. Here, we present details of the superfluid response in the coverage regime immediately below third-layer promotion. A scaling analysis of the inferred superfluid fraction shows the characteristic temperature governing the superfluid response to decrease, approaching zero near the coverage at which simulations predict the second layer to form a conventional incommensurate solid.

## Introduction

The possible experimental realisation of a supersolid state of matter in bulk solid $^4 \hbox {He}$ has been the subject of intense recent interest [1, 2]. In a perfect crystal, supersolidity would rely on the incommensurability of that crystal (spontaneous zero-point vacancies free to tunnel through the crystal) [3]. Otherwise, it may be associated with crystalline defects, such as dislocations. In this latter case, potential supersolidity coexists with solid order [4, 5].

The focus of our research has been a study of the second layer of $^4 \hbox {He}$ on graphite, as a potential two-dimensional supersolid. The several motivations for this approach are as follows. Firstly, there is clear evidence, from NMR and heat capacity measurements of atomically layered $^3 \hbox {He}$ films on graphite, of a solid phase at higher coverages in the second layer somewhat below promotion to the third layer. This 2D solid of $S={1}/{2}$ fermions has strong exchange interactions arising from cyclic ring exchange of $^3 \hbox {He}$ atoms, with clear experimental signatures of the frustrated magnetism in the thermodynamic properties [6–10]. Secondly, torsional oscillator measurements on $^4 \hbox {He}$ films on graphite revealed an anomalous response over a narrow coverage range in the second layer [11]. The prime attractions of this system are its highly quantum nature and the strong coverage tunability.

We have performed torsional oscillator measurements of the $^4 \hbox {He}$ film at 33 coverages in the second layer over the temperature range 2 mK–3 K. This extends the study of this system to an order of magnitude lower in temperature and examines it with a significantly finer grid of coverages. We find evidence for a new state of matter, a quantum state with intertwined superfluid and density wave order, reported elsewhere [12].

In the present article, we focus on a narrow coverage range immediately below the second-layer coverage at which promotion to the third layer occurs (the fourth-coverage regime as referred to in [12]). Simulations predict that just before third-layer promotion the second layer forms a 2D solid, incommensurate with the first-layer lattice potential [13, 14]. The central question we address in this report is how the ‘superfluid’ response disappears on approaching that coverage.

This paper is organised as follows: We first summarise some experimental details of the torsional oscillator measurement and thermometry. We then describe the scaling analysis of the inferred superfluid fraction just below promotion to the third layer. We conclude with an outline of key questions and future prospects.

## Experimental Details

The double torsional oscillator, machined (apart from the cell lid) from a single piece of coin silver, had resonant frequencies at low temperatures of 1423 Hz (antisymmetric mode) and 277 Hz (symmetric mode). All measurements reported were taken with the antisymmetric mode, since the symmetric mode had insufficient frequency stability. The substrate consisted of 48 sheets of exfoliated graphite, $130\,\upmu \hbox {m}$ thick, each diffusion bonded onto silver foils $25\,\upmu \hbox {m}$ thick for thermalisation. Each silver foil is diffusion bonded to the base of the upper part of the cell ensuring good thermalisation down to the lowest temperatures of 1 mK and creating a rigid assembly. The mass sensitivity is $-9.33$ mHz/$\hbox {cm}^3$ STP of $^4 \hbox {He}$. The frequency resolution $\varDelta f/f$ in the antisymmetric mode was $2\times 10^{-9}$. The long-term (over more than a year) frequency stability of this mode exceeded $50\,\upmu \hbox {Hz}$.

In order to conveniently measure over a wide temperature range, from 1 mK to 3 K, the oscillator assembly and associated thermometry were mounted on a copper cell plate that was in turn mounted via brass support rods with insulating (vespel) washers to the top of a copper nuclear adiabatic demagnetisation stage. The cell plate was cooled through a weak thermal link, consisting of copper braid. Thermometry to cover the entire temperature range with high precision was provided by a carbon glass thermometer (above 1.3 K), germanium thermometer (50 mK–6 K) (calibrations provided by manufacturers) and a $^3 \hbox {He}$ melting curve thermometer (1–250 mK), using the superfluid A transition as a fixed point.

**Fig. 1:**
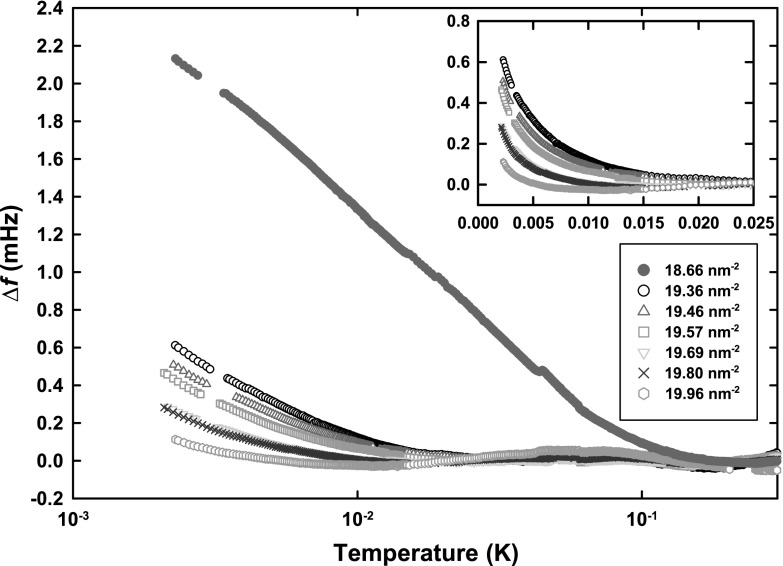
Frequency shift (composite background subtracted and corrected for mass sensitivity) for coverage range between 19.36 and $19.96 \,\hbox {nm}^{-2}$ and the reference coverage of $18.66 \,\hbox {nm}^{-2}$. The *inset* re-plots the same data on linear temperature scale (Color figure online)

For our graphite substrate sample, point-B of a dead volume corrected vapour pressure isotherm [15] corresponded to $15.23\,\hbox {cm}^{3}$. We define our coverage scale by setting the coverage at this point, corresponding to second-layer promotion, to $11.40\,\hbox {nm}^{-2}$. On this scale, we have elsewhere [16, 17] determined the coverage at which promotion to the third layer occurs to be $20.0\,\hbox {nm}^{-2}$. This fiducial point can be used to compare the present results with those of other experiments or the results of simulations. Samples were grown from boiled-off commercial liquid $^4 \hbox {He}$. Possible presence of $^3 \hbox {He}$ at ${\sim }10^{-7}$ concentration level is unlikely to affect robust superfluid signals arising from significant portion (up to tens of per cent) of $^4 \hbox {He}$ atoms in the second layer [12].

## Results

The raw frequency shift data as a function of temperature are first subjected to a composite background subtraction [12]. This background subtracted frequency shift as a function of temperature, for six coverages from 19.36 to $19.96 \,\hbox {nm}^{-2}$, is shown in Fig. 1. Also shown are data for a reference coverage of $18.66 \,\hbox {nm}^{-2}$. From these data, it is apparent that the characteristic temperature governing the onset and temperature dependence of the ‘superfluid’ response decreases with increasing coverage. Over the coverage range under discussion, the maximum temperature at which a superfluid response is resolvable is 15 mK. Given the minimum temperature of the present experiment of 2 mK, the question is how to best access the quantity of most interest: the superfluid frequency shift at $T=0$. This is inaccessible directly, and reliable extrapolation appears challenging.

**Fig. 2:**
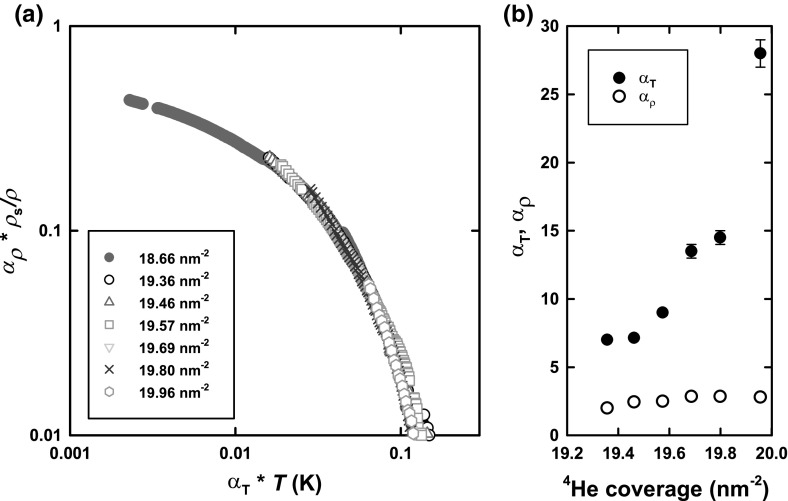
**a** Two-parameter scaling collapse for coverage range between 19.36 and $19.96\,\hbox {nm}^{-2}$ onto the reference coverage of $18.66\,\hbox {nm}^{-2}$. **b** Coverage dependence of the scaling parameters (Color figure online)

However, the key point is that considerable insight can be gained by scaling the data. We note that before scaling the frequency shifts are converted to a superfluid fraction, where calibration is achieved by a measurement of the distinct Kosterlitz–Thouless transition of the fluid layer in a three-layer film.

The striking and distinguishing feature of the data for the coverage ranges 18.17–$18.41\,\hbox {nm}^{-2}$ and 18.50–$19.24 \,\hbox {nm}^{-2}$, reported elsewhere [12], is that they display single-parameter scaling of the form: $\frac{\rho _s(T,n)}{\rho }=\frac{\varDelta (n)}{T_0}f(T/\varDelta (n))$. Here *n* is the second-layer density, and $T_0$ is chosen so that $f(0)=1$, and so $\frac{\rho _s(T=0,n)}{\rho }=\frac{\varDelta (n)}{T_0}$. The superfluid density is then described over a range of coverages by a universal function, with its temperature dependence governed by a single-energy scale $\varDelta (n)$, which depends on layer density. Essentially this means that both the magnitude of the superfluid response and the characteristic temperature governing that response scale between coverages by the same factor.

However for the coverage range of primary interest in this report, from 19.36 to $19.96 \,\hbox {nm}^{-2}$, collapse can only be achieved by two-parameter scaling: ${\alpha _\rho }\frac{{{\rho _s}}}{\rho } = Af\left( {\frac{{{\alpha _T}T}}{\varDelta }} \right)$. In other words, the magnitude of the superfluid response and the characteristic temperature governing that response scale between coverages by different factors. The scaled data are shown in Fig. 2a. The procedure is to plot the superfluid fraction vs. temperature on a log–log plot and determine the scaling factors for each variable which collapse the plots. The data are scaled to coverage $18.66 \,\hbox {nm}^{-2}$. The scale factors are shown in Fig. 2b. The results indicate that while the magnitude of the ‘superfluid’ response does not change significantly with increasing coverage, the characteristic temperature approaches zero approximately linearly. It should be noted that this behaviour is not compatible with classical two-phase coexistence. This is the main result we wish to highlight.

**Fig. 3:**
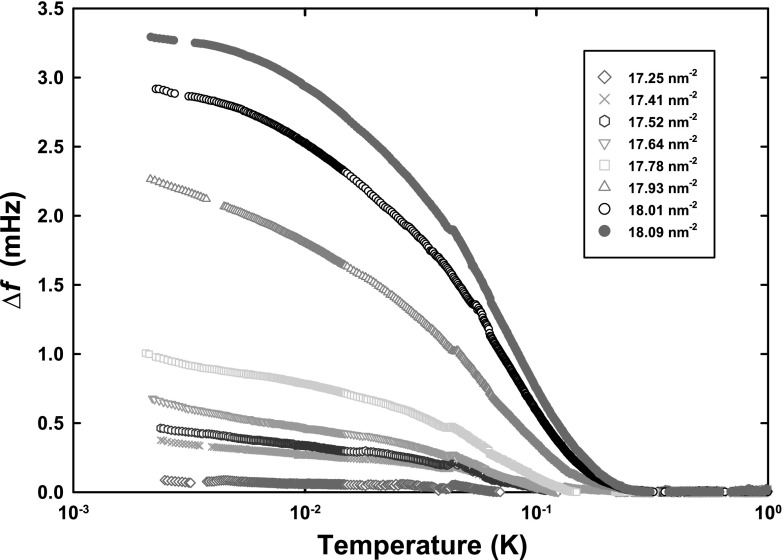
Frequency shift (composite background subtracted and corrected for mass sensitivity) for coverage range between 17.25 and $18.01\,\hbox {nm}^{-2}$ and the reference coverage of $18.09\,\hbox {nm}^{-2}$ (Color figure online)

**Fig. 4:**
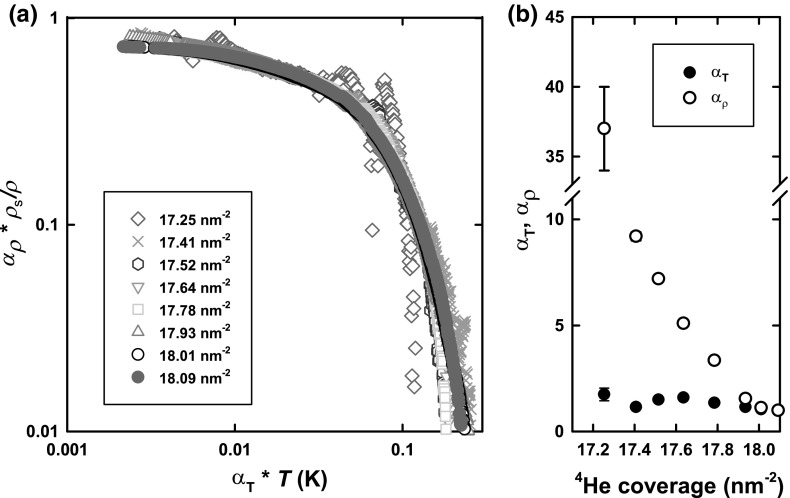
**a** Two-parameter scaling collapse for coverage range between 17.25 and $18.01\,\hbox {nm}^{-2}$ onto the reference coverage of $18.09\,\hbox {nm}^{-2}$. **b** Coverage dependence of the scaling parameters (Color figure online)

To reinforce this point, we contrast with the two-parameter scaling of the data over the coverage range 17.25–$18.09 \,\hbox {nm}^{-2}$, shown in Fig. 3. We believe that this coverage range corresponds to liquid–solid coexistence. The scaled data over this coverage range are shown in Fig. 4a and the scaling parameters in Fig. 4b. Clearly, in this case, the characteristic temperature governing superfluid onset varies only weakly with coverage, while the magnitude of the superfluid response increases with two approximately linear segments. This behaviour is broadly consistent with two-phase coexistence.

It is worth pointing out that, perhaps surprisingly, in this ‘two-phase coexistence’ the non-superfluid phase is the uniform liquid phase. The suppression of superfluidity in a fluid monolayer subject to a periodic potential was previously observed on a graphite substrate with various HD pre-platings [18]. It should also be noted that the uniform fluid layer exists over a relatively narrow coverage range: between an instability at lower densities with respect to 2D condensation, and an instability at higher densities towards a solid-like phase.

In summary, over the coverage range 19.36–$19.96\,\hbox {nm}^{-2}$, we observe unusual two-parameter scaling of the superfluid-like response, in which the characteristic temperature governing that response decreases with increasing coverage, while the magnitude of the superfluid-like response does not change significantly. On our coverage scale $19.9 \,\hbox {nm}^{-2}$ corresponds to that at which the second layer is agreed to form an incommensurate solid. The structure of the second layer in the narrow coverage range immediately preceding this point is unknown.

For more details on comparison of the present torsional oscillator results with second-layer phase diagrams proposed on the basis of heat capacity measurements, and the mapping of our coverage scale to those used in these works, we refer the reader to [12].

## Conclusion

In an earlier report [12], we identified a new state with intertwined superfluid and density wave order in the second layer, with two distinct regimes, corresponding to coverage ranges 18.17–$18.41 \,\hbox {nm}^{-2}$ and 18.50–$19.24 \,\hbox {nm}^{-2}$. In each of these regimes, data collapse is achieved by single-parameter scaling. The interpretation is based on an analysis of the temperature dependence of the superfluid fraction, and its scaling behaviour, as well as the absence of Kosterlitz–Thouless transition. In this regime, the scale of the superfluid response indicates that superfluidity is a property of the entire layer. It is not a defect driven phenomenon, as is the case with scenarios in bulk solid $^4 \hbox {He}$. Rather, it is a consequence of a new state in which superfluidity and density wave order are quantum entangled.

In the results presented here, over a narrow coverage range 19.36–$19.96\,\hbox {nm}^{-2}$, approaching the formation of an incommensurate solid, we find the unusual two-parameter scaling discussed above. One possible interpretation is that the 2D supersolid behaviour of a monolayer of $^4 \hbox {He}$ (the second layer of $^4 \hbox {He}$ on graphite) persists at $T=0$ even in the putative incommensurate solid phase. We believe that the composite background subtraction is sufficiently robust that this is a genuine effect. A more precise determination of this effect, for example by extending measurements to even lower temperature, remains a challenge for the future.

Recent measurements of a series of heat capacity maxima over a fine grid of coverages [19] do provide evidence for transitions in the structure factor and support our torsional oscillator measurements. All these results reinforce the urgent need for direct determination of film structure, to better understand the interplay of structure and superfluidity.
